# Associations between Components of Metabolic Syndrome and Demographic, Nutritional, and Lifestyle Factors

**DOI:** 10.1155/2024/8821212

**Published:** 2024-01-19

**Authors:** Layne Christina Benedito de Assis Lima, Séphora Louyse Silva Aquino, Aline Tuane Oliveira da Cunha, Talita do Nascimento Peixoto, Severina Carla Vieira Cunha Lima, Karine Cavalcanti Maurício Sena-Evangelista, Josivan Gomes Lima, Lucia Fátima Campos Pedrosa

**Affiliations:** ^1^Postgraduate Program in Nutrition, Federal University of Rio Grande do Norte, Av. Salgado Filho, 3000-Lagoa Nova, Natal 59078970, RN, Brazil; ^2^Postgraduate Program in Health Sciences, Federal University of Rio Grande do Norte, Av. Cordeiro de Farias s/n, Natal 59012-570, RN, Brazil; ^3^Collaborative Researcher in Postgraduate Program in Nutrition, Federal University of Rio Grande do Norte, Av. Salgado Filho, 3000-Lagoa Nova, Natal 59078970, Brazil; ^4^Department of Nutrition, Federal University of Rio Grande do Norte, Av. Salgado Filho, 3000-Lagoa Nova, Natal, RN 59078-970, Brazil; ^5^Department of Clinical Medicine, Endocrine Unit, Federal University of Rio Grande do Norte, Av. Nilo Peçanha 620, Petrópolis, Natal, RN 59010-180, Brazil

## Abstract

**Objectives:**

To evaluate the associations between individuals with and without changes in components of metabolic syndrome (MetS) and demographic, nutritional, and lifestyle factors.

**Methods:**

A cross-sectional study was conducted with 224 individuals followed-up at a public hospital in Northeast Brazil. We used National Cholesterol Education Program-Adult Treatment Panel III (NCEP) criteria to diagnose MetS. We assessed components of MetS as dependent variables, while sex, age, food consumption, smoking, alcohol intake, physical activity, anthropometric parameters, and sleep hours were independent variables.

**Results:**

Comparing individuals with and without changes in components of MetS, the logistic regression models revealed that female sex was predictive of increased waist circumference and low HDL-c levels while advanced age was predictive of increased blood pressure and blood glucose levels. BMI emerged as a predictor for waist circumference and a protective factor for triglyceride levels. In addition, potassium intake, physical activity, and sleep duration were protective against decreased HDL-c, elevated triglyceride, and elevated blood pressure levels, respectively.

**Conclusion:**

This study demonstrated that sex, age, BMI, dietary potassium intake, physical activity, and hours of sleep are factors to be targeted in public health actions for prevention and treatment of MetS.

## 1. Introduction

Nowadays, in about 20 countries around the world, the risk of dying prematurely from noncommunicable diseases (NCDs) is higher than dying from infectious and parasitic diseases, maternal and perinatal conditions, and nutritional deficiencies combined. In 2016, an estimated 40.5 million (71%) of the 56.9 million deaths worldwide were due to NCD. The leading causes of NCD deaths (80%) were cancer, cardiovascular diseases, chronic respiratory diseases, and diabetes [1].

Metabolic syndrome (MetS) is a NCD defined as the cluster of cardiometabolic risk factors such as obesity and type 2 diabetes (T2DM). MetS prevalence is estimated at around a quarter of the world population [2]. Previous hypotheses suggested that MetS is initiated by insulin resistance. Interactions between genetic, environmental, and lifestyle factors results in the inherent complexity of preventing and monitoring MetS [3, 4].

Western dietary patterns deemed unhealthy and high alcohol consumption are considered cardiovascular risk factors [5]. A sedentary lifestyle has been associated with the risk of developing T2DM, hypercholesterolemia, and MetS. Smoking is also a risk factor for MetS, associated with HDL-c, increased waist circumference, and high concentrations of triglycerides. In addition, general sleep quality has a positive association with MetS [6].

Identifying the predicting factors for each altered component of the MetS can be an assertive alternative in clinical care due to various MetS phenotypes that gather cardiovascular risk factors. Therefore, this study evaluates the association between components of MetS and demographic, nutritional, and lifestyle factors to identify predictive and protective factors associated with each component of the MetS.

## 2. Methods

### 2.1. Study Design

We carried out a cross-sectional study from 2013 to 2019 with individuals aged between 19 and 77, diagnosed with MetS, followed-up at the Onofre Lopes University Hospital (HUOL), Natal, Northeast Brazil. The Research Ethics Committee of the HUOL approved the study under CAAE number 13699913.7.0000.5292. National Cholesterol Education Program-Adult Treatment Panel III (NCEP) criteria [7] were used to diagnose MetS. We excluded individuals with type 1 diabetes (T1DM) or T2DM using insulin or using glucocorticoids for the past three months, kidney (MDRD < 60 mL/min) or hepatic changes (transaminases three times over the upper normal limit), decompensated heart failure; pregnancy or lactation, and treatment with antiepileptic drugs or rifampicin from the study. For sample size calculation, multinominal logistic regression models were used, and a total of 220 individuals, including losses, was estimated. Medical records were initially screened and 334 met the study criteria. Among the eligible records, 77 individuals were absent on the collection day and 33 were lost due to abandonment. Therefore, data collection was completed with 224 participants.

### 2.2. Lifestyle Variables

Sleep hours were obtained from the average number hours individuals slept at night. Alcohol intake was assessed based on the quantity and type of alcoholic beverage consumed over a month [8]. The individuals were categorized for smoking as follows: never smoked, nonsmokers, ex-smokers, and smokers [9]. The level of physical activity was evaluated through the Brazilian version of the International Physical Activity Questionnaire [10]. Individuals were classified as sedentary, irregularly active (categories A and B), active, and very active. Irregularly active A category means one who meets at least one of the recommendation criteria in terms of frequency or duration of activity (frequency of 5 days/week or duration of 150 min/week), and irregularly active B has not met any of the recommendation criteria in terms of frequency or duration.

### 2.3. Blood Pressure and Anthropometric Nutritional Status

High blood pressure was defined as systolic pressure ≥130 mmHg and diastolic pressure ≥85 mmHg [7]. The anthropometric evaluation was performed using body mass index (BMI) and waist circumference (WC). The World Health Organization (WHO) cutoff points [11] for BMI classification were used for adults, while the references proposed by the Brazilian Ministry of Health [12] were used for older adults. For the WC classification, WC values >102 cm in men and >88 cm in women were considered for diagnosis [7].

### 2.4. Biochemical Analysis

Fasting blood glucose, total cholesterol, HDL-c, and triglycerides were analyzed using commercial kits from the Wiener lab® (Wiener lab group, Argentina) and an automated CMD800iX1 equipment (Diamond Diagnostics, Holliston, MA, USA). Lipid and blood glucose profiles were classified according to NCEP criteria [7].

### 2.5. Food and Dietary Intake

The 24-hour dietary recall method was applied twice with a time interval between 30 and 45 days. Data on nutritional intake were analyzed using the Virtual Nutri Plus® 2.0 software. The energy and nutrient data were initially adjusted for inter- and intrapersonal variability [13]. The adjustment by energy was made through the residual method [14]. The adequacy of energy, macronutrient, and fiber intake was classified as recommended by the I Brazilian guidelines for diagnosing and treating metabolic syndrome [15]. Micronutrient intake was compared to the estimated average requirement (EAR) or adequate intake (AI) [16–18].

### 2.6. Statistical Analysis

First, mean (standard deviation (SD)) or medians (interquartile interval, *Q*1–*Q*3) were calculated for the descriptive analysis of continuous variables. The normality of the data was tested using the Kolmogorov–Smirnov *Z* test. The Student's *t* test was employed to compare mean values for normally distributed data. For data which were not normally distributed, mean values were compared using the Wilcoxon test. A Chi-squared test was used to verify associations between the categorical variables. Fisher's exact test was used for data with 25% of the expected frequencies lesser than five. The imputation method was applied through the multivariate imputation by chained equation algorithm for missing data of weight, height, systolic and diastolic blood pressure, triglycerides, and sleep hours [19].

The potential predictive or protective factors for the components of MetS were analyzed using univariate logistic regression. Components of MetS (WC, HDL-c, triglycerides, blood glucose, and blood pressure) were included as dependent variables. The independent variables were sex, age, sleep hours, smoking, alcohol intake, physical activity level, BMI, WC, and dietary components. The independent variables included in the logistic regression model were selected according to the Wilcoxon test, Student's *t* test, and Chi-squared distribution (*p* < 0.05).

A logistic regression model with the stepwise (inclusion) method was performed with variables that showed statistically significant differences (*p* < 0.05) in each component of the MetS. We found five final models, namely, (1) WC: sex and BMI, (2) HDL-c: sex and potassium intake, (3) triglycerides: BMI and physical activity, (4) glucose: age, and (5) blood pressure: age and sleep hours. The variance inflation factor test was used to check the absence of multicollinearity from the variables. The odds ratios (ORs) represent the measure of association between exposure to predictive factors compared to alteration or not in the components of MetS. The statistical significance level adopted in all analyses was *p* < 0.05. All analyses were performed using R statistical software (v. 3.5.3).

## 3. Results

The mean age (SD) of the participants, 76.8% of whom were female, was 51 (12) years. Participants were treated with antihypertensive (73.2%), hypoglycemic (39.3%), and lipid-lowering agents (37%). The median systolic and diastolic blood pressures were above recommended parameters. Most individuals reported being moderately to very active, not smoking, and not consuming alcohol. Body mass index (BMI), triglyceride, and blood glucose values were increased while HDL-c levels were decreased.

Comparing individuals with and without changes in components of MetS, females more often presented with WC changes. Monthly alcohol intake was more frequent in individuals with high blood pressure while hours of sleep were significantly shorter among individuals with high blood pressure (Table 1). Low HDL-c levels were most frequent in females and individuals with between one and four alcoholic beverages per month. High triglyceride levels were more frequently observed in individuals in the irregularly active B group. Increased blood glucose levels were most prevalent in females and smokers (Table 2).

Comparing the daily dietary energy and nutrient intake and the relationship with components of MetS, energy intake was lower in individuals with high blood pressure (Table 3). Individuals with low HDL-c levels consumed smaller quantities of selenium and larger quantities of potassium. Higher copper and potassium intake was observed among individuals with higher blood glucose levels (Table 4). Among components of MetS, the percentage of calories intake from carbohydrates in relation to the total caloric intake of the diet was adequate, except in individuals without changes in WC and blood pressure (48.8%). Protein intake was above the recommended level for all components (between 15.6 and 17.6%); meanwhile, fiber intake (12–13.5 g/day) was below recommended levels. Total fat values were within the normal range (between 21.7 and 27.8%) [15] (Tables 3 and 4).

Logistic regression models revealed that female sex was predictive of increased WC and low HDL-C levels while advanced age was predictive of increased blood pressure and blood glucose levels. BMI emerged as a predictive and protective factor for waist circumference and triglyceride levels, respectively. Finally, potassium intake, physical activity, and sleep duration were protective against decreased HDL-C, elevated triglyceride, and elevated blood pressure levels, respectively (Table 5).

## 4. Discussion

This study showed that females were more likely to have increased WC and low HDL-c among patients with MetS. Advanced age was a predictive factor for arterial hypertension and glycemia, BMI was a predictive factor for WC and a protective factor for triglycerides, sleeping hours showed positive effects on arterial hypertension, dietary potassium intake was associated with low HDL-c, and physical activity was a protective factor for increased triglycerides in this population.

Different findings of the prevalence of MetS in the sexes have been reported [20, 21], but women over 50 are more likely to develop MetS. Insulin resistance increased abdominal obesity, and reduced HDL-c are frequent metabolic disorders after menopause, in addition to other disorders related to diet and psychosocial changes [21]. Age has been found to be a predictive factor for hypertension, which can be explained by hemodynamic changes that take place with age in the vascular system that affect blood pressure, such as increased stiffness and pressure in the arteries [22]. Advanced age is also a risk factor for T2DM. Glucose intolerance increases with age due to body fat accumulation, eating habits changes, and physical inactivity [23].

The association between BMI and WC highlights the importance of BMI as an anthropometric indicator in diagnosing MetS in clinical practice [24]. However, the inverse association observed between BMI and triglycerides should be cautiously interpreted since BMI cannot differentiate between lean mass and fat and cannot measure visceral fat accurately. Some biases arising from the intrinsic characteristics of the population must be considered. For example, in Table 2, the categorization of participants according to BMI and hypertriglyceridemia values (yes/no) shows that in the “no” category, the distribution of BMI results was more heterogeneous (29.4–39.9) in addition to presenting the highest values. BMI also has limitations regarding significant associations with metabolic markers, including those related to lipid profiles [25]. In addition, 37% of the sample used lipid-lowering agents, and it is attributed that individuals with higher BMI were targets of drug interventions focused on the lipid profile, whose effects on metabolic control are practical, regardless of weight loss.

Our finding of a negative association between sleeping hours and blood pressure is in line with current evidence on the impact of sleep on MetS [26, 27]. Sleep duration influences blood pressure through hormonal imbalances, increased adiposity, metabolic dysfunction, and circadian rhythm disturbances [28]. In addition, waking up late can also affect health due to high concentrations of cortisol, which can increase blood glucose, heart rate, and blood pressure [29]. Thus, the results found in this study can also be extended to assess sleep quality and latency in individuals with MetS.

In the present study, patients with high blood pressure had lower energy intake than those with normal blood pressure. It is essential to highlight that caloric intake is not the only indicator of diet quality that influences blood pressure, and it is crucial to consider this factor together with the quality of the nutrients consumed. The effects of dietary composition on blood pressure are influenced by several mechanisms, in particular, mediated by body weight loss, attenuation of systemic inflammation, increased insulin sensitivity, and antihypertensive effects inherent to certain isolated nutrients [30]. In addition, during the clinical follow-up of these patients with high blood pressure, sodium restriction in the diet is emphasized, possibly leading to a reduction in the consumption of processed foods rich in sodium, which, in turn, may influence the total calorie intake.

We found a low consumption of dietary fiber (between 12 and 13 g/day), and minimum consumption of 25 g per day is recommended to improve glycemic control and attenuate postprandial hyperglycemia [31]. In this context, the difference observed between total fiber intake and hyperglycemia is insignificant for application in dietary guidelines. Copper intake was higher in individuals with hyperglycemia. Still, this finding would need to be further explored in models of association with glycemic control markers, but the variable was not classified to be included in logistic regression. However, a study conducted with adult subjects without MetS demonstrated by multiple regression models that dietary copper intake was inversely associated with fasting glucose [32]. Cooper has a pro-oxidant role in the metal-catalyzed formation of free radicals. It has been explored in diabetes due to its relationship with glycemic control [33].

Potassium intake was found to be a protective factor for low HDL-c, an uncommon finding in the literature. However, we recognize the limitation of discussing dietary potassium intake without associating it with biochemical markers and urinary excretion to elucidate probable mechanisms of the relationship with HDL-c. Low dietary potassium intake may increase the risk of chronic diseases such as T2DM and cardiovascular disease [34, 35]. Our population's average daily potassium intake was lower than the adequate intake (AI) values of 3 400.0 mg for men and 2 600.0 mg for women [18].

A lower selenium intake was observed in individuals with low HDL-c. Selenium, as an essential trace element, plays an important role in lipid metabolism protecting against damage caused by oxidative stress. It is believed that adequate selenium intake may reduce the risk of chronic diseases from oxidative and inflammatory imbalances associated with MetS [36, 37].

Our results reinforce the need of practicing physical activity for individuals with MetS, especially those with hypertriglyceridemia. The benefits of physical activity for individuals with MetS include improved body composition, cardiovascular health, and metabolic profile [38]. WHO recommends that adults perform at least 150–300 minutes of moderate-intensity aerobic physical activity or at least 75–150 minutes of vigorous-intensity aerobic physical activity or an activity-equivalent combination of moderate or vigorous exercise throughout the week for substantial health benefits [39].

The lack of association observed between blood pressure and physical activity levels can be attributed to the complex multifactorial interaction to be considered in the approach to blood pressure, such as genetic/epigenetic, environmental, and social [40], which masks the significance of the level of physical activity as the only determining factor of arterial hypertension. It is also noteworthy that the participants received specific guidance for treating high blood pressure, which may have influenced the association of some variables. Another factor to be considered is the instrument used (International Physical Activity Questionnaire (IPAQ)), a short version validated in Brazil, which probably overestimated the level of physical activity [41]. In addition, the complexity in the interpretation of specific questions, such as the distinction between vigorous and moderate activities, as well as the difficulty in quantifying sedentary activities [10], may have impacted the accuracy of the measurements.

We found no statistical difference between smoking and MetS; however, our results align with other studies [42, 43]. By the way, life-course cigarette smoking was associated with increased odds of MetS, especially among individuals aged <70 years [44]. It is crucial to consider that the history of smoking is a complex and multidimensional phenomenon, encompassing aspects such as duration, intensity of the habit, and time since cessation across life course. Many researchers adopt simplified approaches to avoid the analytical challenges posed by this multidimensionality, focusing on a single factor of smoking in their studies. However, this simplification can result in the loss of crucial information related to other dimensions of smoking. Even if we choose to model several smoking-related variables simultaneously, this can induce multicollinearity or extremely unstable estimates [45]. In our study, we decided to categorize individuals into four distinct groups concerning smoking: “never smoked,” “nonsmokers,” “ex-smokers,” and “smokers” according to Brazilian consensus [9]. We recognize that this approach represents a simplification of the complexity of smoking by focusing on broad categories.

This study presents some limitations, such as the cross-sectional design, the inherent complexity of the evaluation of food intake, and the assessment of sleep duration without assessment of sleep quality and latency. A strong point of the study is the investigation of biological, nutritional, and lifestyle factors related to isolated components of MetS, making it possible to identify suggestions of clinical actions.

This study demonstrated that sex, age, BMI, dietary potassium intake, physical activity, and hours of sleep are factors to be targeted in public health actions for prevention and treatment of MetS. Furthermore, the knowledge of predictive and protective factors for each MetS component is also fundamental to guide the monitoring of MetS with a focus on phenotypes most frequent in each population.

## Figures and Tables

**Table 1:** Comparing demographic, nutritional, and lifestyle factors and changes in waist circumference and blood pressure in patients with metabolic syndrome.

Factors	Increased waist circumference^a^			Elevated blood pressure^b^		*p*
	Yes	No	*P*	Yes	No	
*Sex* ^c^						
Male	30 (57.7)	22 (42.3)	<0.001	35 (67.3)	17 (32.7)	
Female	167 (97.1)	05 (2.9)		129 (75.0)	43 (25.0)	0.272

BMI (kg/m^2^)^d^	32.7 (29.5–39.1)	25.8 (24.1–27.5)	<0.001	32.0 (28.2–38.0)	31.9 (28.4–37.0)	0.992

*Smoking* ^c^						
Never smoked	86 (91.5)	08 (8.5)		69 (73.4)	25 (26.6)	
Nonsmokers	34 (81.0)	08 (19.0)		28 (66.7)	14 (33.3)	
Ex-smoker	63 (86.3)	10 (13.7)	0.302	55 (75.3)	18 (24.7)	0.696
Smokers	14 (93.3)	01 (6.7)		12 (80.0)	03 (20.0)	

*Alcohol consumption/month* ^c^						
Nonconsumer	101 (87.1)	15 (12.9)		90 (77.6)	26 (22.4)	
1 drink	50 (90.9)	05 (9.1)		43 (78.2)	12 (21.8)	
1–4 drinks	20 (90.9)	02 (9.1)	0.751	11 (50.0)	11 (50.0)	**0.028**
>5 drinks	26 (83.9)	05 (16.1)		20 (64.5)	11 (35.5)	

*Physical activity* ^c^						
Sedentary	17 (73.9)	06 (26.1)		14 (60.9)	09 (39.1)	
Irregularly active A	43 (91.5)	04 (8.5)		34 (72.3)	13 (27.7)	
Irregularly active B	31 (86.1)	05 (18.5)	0.149	27 (75.0)	09 (25.0)	0.541
Active/very active	106 (89.8)	12 (10.2)		89 (75.4)	29 (24.6)	
Sleep hours^d^	7.0 (6.0–8.0)	7.0 (6.0–8.0)	0.745	7.0 (6.0–8.0)	8.0 (6.0–8.0)	**0.002**

^a^Reference values: >102 cm for men and >88 cm for women (NCEP, 2002); ^b^reference values: systolic blood pressure ≥130 mmHg or diastolic blood pressure ≥85 mmHg (NCEP, 2002); ^c^data presented as *n* (%); ^d^data presented as medians (*Q*1 = quartile 1 or percentile 25; *Q*3 = quartile 3 or percentile 75). BMI: body mass index. Significant values are given in bold (*p* < 0.05, Chi-square test, Fisher's exact, and Wilcoxon test).

**Table 2:** Comparing demographic, nutritional, and lifestyle factors and changes in HDL-c, triglycerides, and blood glucose in patients with metabolic syndrome.

Factors	Low HDL-c^a^		*p*	Hypertriglyceridemia^b^		*p*	Hyperglycemia^c^		*p*
	Yes	No		Yes	No		Yes	No	
*Sex* ^d^									
Male	36 (69.2)	16 (30.8)	0.008	36 (69.2)	16 (30.8)	0.151	45 (86.5)	07 (13.5)	**0.013**
Female	147 (85.5)	25 (14.5)		100 (58.1)	72 (41.9)		119 (69.2)	53 (30.8)	
BMI (kg/m^2^)^e^	32.0 (28.1–37.0)	31.6 (28.7–39.1)	0.973	31.5 (27.9–32.7)	33.2 (29.4–39.9)	0.015	32.0 (28.2–38.0)	32.0 (29.0–37.0)	0.738

*Smoking* ^d^									
Never smokers	79 (84.0)	15 (16.0)	0.784	55 (58.5)	39 (41.5)	0.747	62 (66.0)	32 (34.0)	**0.032**
Nonsmokers	35 (83.3)	07 (16.7)		26 (61.9)	16 (38.1)		28 (66.7)	14 (33.3)	
Ex-smoker	57 (78.1)	16 (21.9)		44 (60.3)	29 (39.7)		61 (84.6)	12 (16.4)	
Smokers	12 (80.0)	03 (20.0)		11 (73.3)	04 (26.7)		13 (86.7)	02 (13.3)	

*Alcohol consumption/month* ^d^									
Don't consume	99 (85.3)	17 (14.7)	0.018	70 (60.3)	46 (39.7)	0.564	82 (70.7)	34 (29.3)	0.115
1 drink	46 (83.6)	09 (16.4)		30 (54.5)	25 (45.5)		42 (25.6)	13 (21.7)	
1–4 drinks	19 (86.4)	03 (13.6)		15 (68.2)	07 (31.8)		13 (59.1)	09 (40.9)	
>5 drinks	19 (61.3)	12 (38.7)		21 (67.7)	10 (32.3)		27 (87.1)	04 (12.9)	

*Physical activity* ^d^									
Sedentary	19 (82.6)	04 (17.4)	0.465	17 (73.9)	06 (26.1)	0.003	19 (82.6)	04 (17.4)	0.345
Irregularly active A	42 (89.4)	05 (10.6)		34 (72.3)	13 (27.7)		35 (74.5)	12 (25.5)	
Irregularly active B	28 (77.8)	08 (22.2)		27 (75.0)	09 (25.0)		29 (80.6)	07 (19.4)	
Active/very active	94 (79.7)	24 (20.3)		58 (48.1)	60 (50.9)		81 (68.6)	37 (31.4)	
Sleep hours^e^	7.0 (6.0–8.0)	7.0 (6.0–7.7)	0.43	7.0 (6.0–8.0)	7.0 (6.0–8.0)	0.224	7.0 (6.0–8.0)	7.0 (6.0–8.0)	0.367

^a^Reference values: <40 mg/dL for men and <50 g/dL for women (NCEP, 2002), ^b^reference values: ≥150 mg/dL or use of lipid-lowering agents (NCEP, 2002), ^c^reference values: ≥100 mg/dL or use of hypoglycemic agents (NCEP, 2002), ^d^Data presented as *n* (%), and ^e^data presented as median (*Q*1 = quartile 1 or percentile 25; *Q*3 = quartile 3 or percentile 75). Significant values are given in bold (*p* < 0.05, Chi-square test, Fisher's exact, and Wilcoxon test).

**Table 3:** Comparing energy and nutrient intake with waist circumference and blood pressure in patients with metabolic syndrome.

Intake/day	Increased waist circumference^a^		*p*	Elevated blood pressure^b^		*p*
	Yes	No		Yes	No	
Energy (kcal)^c^	1429.0 (1228.6−1429.4)	1621.0 (1188.0−2174.0)	0.104	1371.4 (1179.9–1681.2)	1599.5 (1398.0–1833.5)	**0.002**
Carbohydrate (g)^c^	195.8 (185.0–213.6)	197.8 (187.2–215.9)	0.679	196.4 (185.6–210.0)	195.2 (183.6–218.2)	0.516
Protein (g)^d^	63.0 (11.0)	63.6 (11.2)	0.801	63.2 (11.0)	62.8 (11.1)	0.807
Total fat (g)^d^	42.9 (8.1)	39.2 (9.0)	0.054	42.5 (8.6)	42.2 (7.5)	0.83
Total fiber (g)^c^	12.3 (10.1–14.1)	13.5 (11.3–15.5)	0.071	12.3 (10.3–14.6)	12.1 (10.1–14.0)	0.588
Cholesterol (mg)^c^	194.8 (153.0–239.5)	182.9 (161.5–208.6)	0.765	194.2 (153.8–237.0)	185.5 (154.1–242.7)	0.685
Vitamin A (*µ*g)^c^	564.5 (370.1–783.6)	493.2 (295.7–673.5)	0.355	564.9 (367.5–787.8)	548.7 (401.3–727.3)	0.719
Vitamin C (mg)^c^	81.5 (55.0–107.8)	104.3 (57.3–125.9)	0.154	81.7 (55.1–111.0)	86.3 (53.7–108.9)	0.914
Vitamin E (mg)^c^	11.4 (8.9–14.4)	11.6 (8.6–14.7)	0.788	11.3 (8.9–14.5)	11.7 (8.9–14.1)	0.634
Copper (mg)^c^	0.84 (0.73–0.95)	0.83 (0.78–0.93)	0.827	0.8 (0.8−0.10)	0.8 (0.7–0.9)	0.286
Magnesium (mg)^d^	169.6 (28.8)	175.1 (24.8)	0.304	170.1 (27.6)	170.7 (30.5)	0.901
Selenium (*µ*g)^d^	57.0 (15.5)	52.9 (14.8)	0.187	53.4 (14.8)	53.4 (17.2)	0.982
Zinc (mg)^c^	6.2 (5.3–7.6)	6.2 (5.8–7.6)	0.534	6.3 (5.3–7.8)	5.9 (5.4–7.2)	0.2
Potassium (mg)^c^	1683.6 (1411.6–1941.7)	1751.2 (1552.5–2007.2)	0.216	1688.9 (1439.0–1938.5)	1684.9 (1418.8–1970.1)	0.862
Sodium (mg)^c^	2022.2 (1698.4–2315.4)	2073.4 (1819.4–2351.6)	0.621	2002.7 (1718.1–2308.7)	2113.7 (1833.5–2462.6)	0.118

^a^Reference values: >102 cm for men and >88 cm for women (NCEP, 2002); ^b^Reference values: systolic blood pressure ≥130 mmHg or diastolic blood pressure ≥85 mmHg (NCEP, 2002); ^c^Data presented as median (*Q*1 = quartile 1 or percentile 25; *Q*3 = quartile 3 or percentile 75); ^d^Data presented as mean (standard deviation); significant values are given in bold (*p* < 0.05, Wilcoxon test and Student's *t* test).

**Table 4:** Comparing energy and nutrient intake with changes in HDL-c, triglyceride, and blood glucose levels in patients with metabolic syndrome.

Intake/day	Low HDL-c^a^		*p*	Hypertriglyceridemia^b^		*p*	Hyperglycemia^c^		*p*
	Yes	No		Yes	No		Yes	No	
Energy (kcal)^d^	1506.1 (1228.0–1716.1)	1422.8 (1236.0–1777.0)	0.466	1463.6 (1216.9–1770.3)	1427.7 (1233.6–1689.9)	0.823	1456.6 (1249.3–1704.0)	1420.1 (1172.8–1773.7)	0.766
Carbohydrate (g)^d^	199.2 (185.1–213.8)	194.4 (185.1–216.8)	0.831	197.1 (186.9–214.1)	194.3 (183.6–213.8)	0.687	196.8 (185.6–214.7)	194.5 (184.2–210.5)	0.889
Protein (g)^e^	62.6 (11.1)	65.6 (10.3)	0.099	62.6 (10.5)	64.0 (11.8)	0.363	63.7 (10.8)	61.6 (11.5)	0.216
Total fat (g)^e^	42.6 (8.3)	41.7 (8.1)	0.534	42.9 (8.2)	41.7 (8.3)	0.279	41.9 (8.4)	43.7 (7.8)	0.137
Total fiber (g)^d^	12.3 (10.2–14.3)	12.1 (11.0–14.6)	0.883	12.3 (10.2–14.0)	12.3 (2.6–3.9)	0.949	12.3 (10.5–15.0)	12.0 (9.6–13.5)	**0.022**
Cholesterol (mg)^d^	192.3 (153.6–238.8)	190.3 (158.2–237.1)	0.959	192.3 (154.5–242.8)	197.9 (153.0–230.6)	0.949	189.3 (153.7–235.1)	199.9 (154.0–250.0)	0.36
Vitamin A (*µ*g)^d^	555.9 (368.3–750.6)	564.5 (403.0–894.7)	0.404	550.9 (354.8–757.8)	563.7 (410.9–801.1)	0.374	565.2 (366.1–811.4)	545.7 (422.6–726.0)	0.636
Vitamin C (mg)^d^	81.5 (55.1–114.5)	88.9 (53.9–101.4)	0.775	81.7 (57.3–113.2)	83.8 (51.7–108.5)	0.626	84.9 (55.1–114.3)	74.5 (53.4–106.3)	0.21
Vitamin E (mg)^d^	11.6 (8.9–14.5)	10.9 (8.9–14.3)	0.573	11.6 (9.0–14.6)	10.8 (8.6–14.3)	0.211	11.4 (8.9–14.4)	11.5 (8.9–14.5)	0.754
Copper (mg)^d^	0.8 (0.7–1.0)	0.9 (0.7–0.9)	0.424	0.8 (0.7–0.9)	0.8 (0.7–1.0)	0.946	0.9 (0.8–1.0)	0.8 (0.7–0.9)	**0.038**
Magnesium (mg)^e^	168.9 (28.6)	176.6 (26.7)	0.105	169.8 (28.1)	171.0 (28.9)	0.766	172.1 (28.6)	165.3 (27.3)	0.107
Selenium (*µ*g)^e^	52.3 (15.5)	58.4 (14.4)	**0.018**	53.8 (14.8)	52.7 (16.5)	0.633	53.1 (15.5)	54.2 (15.4)	0.642
Zinc (mg)^d^	6.2 (5.4–7.6)	6.2 (5.0–7.4)	0.433	6.2 (5.5–7.5)	6.3 (5.3–7.7)	0.924	6.2 (5.3–7.7)	6.0 (5.4–7.2)	0.486
Potassium (mg)^d^	1838.9 (1412.6–1940.0)	1649.3 (1581.3–2045.1)	**0.037**	1690.7 (1435.7–1925.5)	1674.3 (1474.3–1996.6)	0.565	1728.3 (1502.5–1988.0)	1549.3 (1340.1–1895.2)	**0.015**
Sodium (mg)^d^	2018.8 (1748.5–2317.8)	2080.3 (1731.5–2343.5)	0.656	2035.2 (1783.4–2349.0)	2008 (1632.5–2301.6)	0.23	1994.9 (1710.0–2309.2)	2117.7 (1797.1–2349.0)	0.384

^a^Reference values: <40 mg/dL for men and <50 mg/dL for women (NCEP, 2002); ^b^reference values: ≥150 mg/dL or use of lipid-lowering agents (NCEP, 2002); ^c^reference values: ≥100 mg/dL or use of hypoglycemic agents (NCEP, 2002); ^d^data presented as median (*Q*1 = quartile 1 or percentile 25; *Q*3 = quartile 3 or percentile 75); ^e^data presented as mean (standard deviation); significant values are given in bold (*p* < 0.05, Wilcoxon test and Student's *t* test).

**Table 5:** Logistic regression for the prediction of associations among the independent variables and components of metabolic syndrome.

Components	Estimate	SE	*Z*	*p*	OR	95% CI
*Waist circumference*						
Intercept	−20.66	4.409	−4.686	<0.001		
Sex^a^	4.749	0.976	4.865	<0.001	1.155	2.177; 1.087
BMI	0.717	0.154	4.662	<0.001	2.048	1.583; 2.929

*HDL-c*						
Intercept	2.539		2.812	0.005	0.903	
Sex	0.933	0.374	2.495	0.013	2.543	1.208; 5.276
Potassium intake	−0.001	0.001	−2.053	0.04	0.999	0.998; 0.999

*Triglycerides*						
Intercept	2.903	0.894	3.247	0.001		
BMI	−0.053	0.021	−2.543	0.011	0.947	0.908; 0.987
Irregularly active A^b^	−0.117	0.587	−0.199	0.842	0.89	0.267; 2.746
Irregularly active B^b^	−0.011	0.624	−0.017	0.986	0.989	0.279; 3.330
Active/very active^b^	−1.158	0.523	−2.216	0.026	0.314	0.104; 0.834

*Blood glucose*						
Intercept	−1.986	0.663	−2.993	0.003		
Age	0.061	0.013	4.48	<0.001	1.063	1.036; 1.09

*Blood pressure*						
Intercept	0.427	1.056	0.444	0.686		
Age	0.049	0.013	3.937	<0.001	1.05	1.024; 1.079
Sleep hours	−0.258	0.111	−2.078	0.02	0.772	0.617; 0.956

SE, standard error; OR, odds ratio; CI, confidence interval; BMI, body mass index; irregularly active category A, meeting at least one of the recommendation criteria (frequency of 5 days/week or duration of 150 min/week); irregularly active category B did not meet any of the recommendation criteria in terms of frequency or duration. ^a^Male sex was used as baseline; ^b^Sedentary lifestyle was used as baseline.

## Data Availability

The datasets used and/or analyzed during the current study are available from the corresponding author on reasonable request.
